# Endoscopic Removal of an Uncommon Iatrogenic Foreign Body from the Maxillary Sinus: A Dental Burr

**DOI:** 10.1155/2020/8861701

**Published:** 2020-12-28

**Authors:** Nafil Arimbrathodi, Waqar Aslam, Abhishek Menon, Ali Ahmad Al Saadi

**Affiliations:** Department of Otolaryngology, Hamad Medical Corporation, Doha, Qatar

## Abstract

Instrument fracture during procedure is not uncommon for dental surgeons, especially in root canal surgeries, usually inside the root canals. In rare instances, high-speed rotary instruments can be fractured and can be dislodged in key anatomical areas of face. In our case report, a high-speed dental burr most probably penetrated the root and was seen in the left maxillary sinus during a likely routine dental procedure. The work-up and endoscopic surgical management of the case is described. Practitioners should be in great care during dental procedures and endodontic treatment to avoid unexpected complications by introducing foreign bodies into maxillary sinus. Any patient presenting with recurrent unilateral facial pain or unilateral sinus symptoms with/without previous history of sinusitis should raise the suspect of a foreign body in the paranasal sinus regardless of any previous history of dental procedure.

## 1. Introduction

Sinus foreign bodies are commonly encountered with ciliary impairment and secondary infection due to their physical and chemical insult to the mucosa. Therefore, the removal of all sinus foreign bodies is recommended, irrespective of whether they produce symptoms or not.

Foreign bodies are occasionally found in the paranasal sinuses [1, 2]. Th common causes for sinus foreign body are following: the entry of material via an oroantral fistula, facial trauma, and iatrogenic causes [2, 3]. Most foreign bodies are pieces of metal, wood, or glass, and they are detected by plain radiography, xeroradiography, computed tomography, magnetic resonance imaging, and ultrasonography [2, 4]. They have seen as a result of complication of a dental procedure [2, 5]. Antral perforation following a dental procedure involving apical surgery of the maxillary molar teeth often creates a pathway for foreign bodies to enter the maxillary sinus [2, 6]. The reported incidence of displaced dental instruments in the maxillary sinus is few [2, 5]. Literature review showed following foreign bodies in maxillary sinus: displaced teeth [7], oral implants [6, 8], gutta-percha points [9], dental burr [5, 10], dental amalgam [11, 12], and impression material [13]. Another case was reported in which patient accidentally inserted a sewing needle into maxillary sinus while trying to clear a dental abscess collection [14]. A wooden toothpick [15] was removed from maxillary sinus in a patient with oroantral fistula which developed after an upper second molar extraction.

In this case report, a high-speed dental burr most probably penetrated the root and was seen in the left maxillary sinus during a likely routine dental procedure. The systematic work-up of the case with endoscopic surgical management is described.

## 2. Case Description

**Figure 1:**
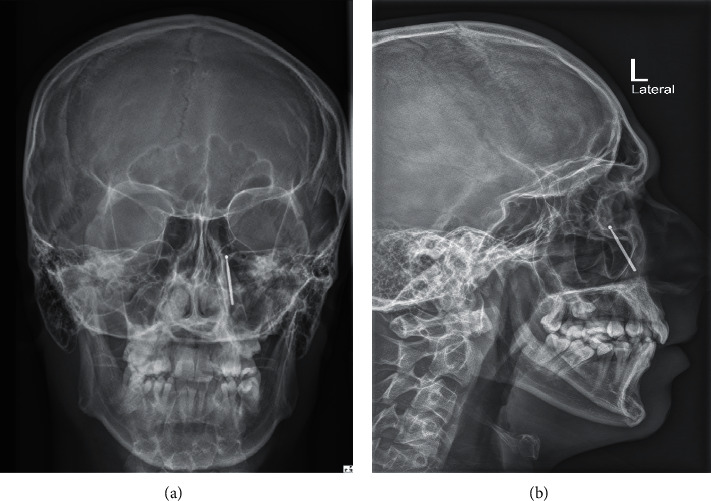
The anterioposterior and lateral view X-ray showing the radio opaque foreign body situated in the left maxillary sinus with head of the foreign body pointing posteriorly.

A 27-year-old male patient presented repeatedly to the primary health care center with complains of left-sided facial pain. The patient gave a history of dental procedure few months back. Due to the recurrent visits, the physician ordered an X-ray Waters' view (Figures 1(a) and 1(b)) which revealed a radio opaque shadow of suspected metallic foreign body in the left maxillary sinus. The patient was then referred to tertiary center.

The patient instead went to a private hospital with the same earlier complaints of left facial pain. He did not show the details of the X-ray findings from the health center visit or about the dental intervention that he underwent few months back. The patient was then sent for an MRI; during the procedure, the patient developed severe facial pain that the MRI had to be abandoned. After which, the patient revealed the X-ray findings to the doctor.

**Figure 2:**
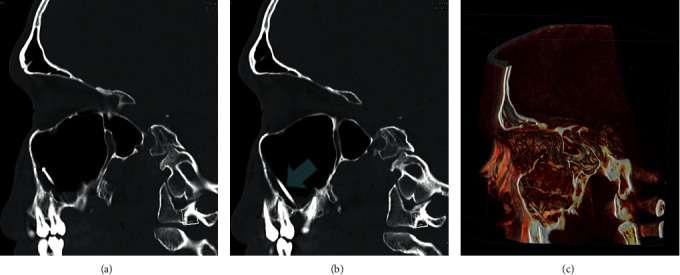
Sagittal cuts of CT sinus showing the head of the foreign body pointing anteriorly, most likely due to the change in position during the MRI. 3D reconstructed CT scan paranasal sinus showing the foreign body.

He was referred to our ENT Department, and clinical examination findings were normal. There was no oroantral fistula. A paranasal sinus CT with 3D reconstruction (Figures 2(a)–2(c)) was ordered, which revealed the dental burr (foreign body) in the left maxillary sinus. It was seen dislodged compared to the X-ray (which was done in the primary health center), with the tip pointing anteriosuperiorly, while the tip was pointing posteriosuperiorly on the X-ray (suggesting the foreign body moved during the MRI).

**Figure 3:**
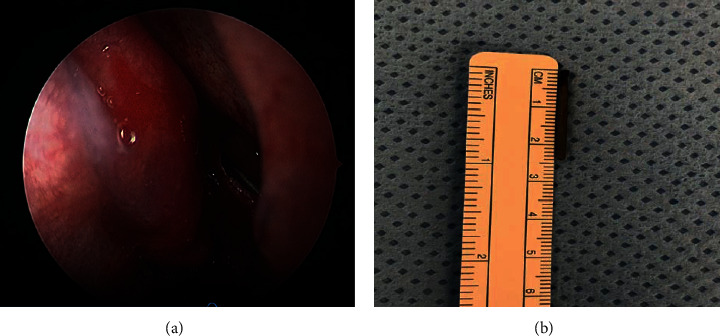
Endoscopic view showing dental burr in maxillary sinus. Foreign body after removal.

The patient was then posted for endoscopic sinus surgery as an outpatient procedure. We did an endoscopic middle meatal antrostomy under navigation protocol. The foreign body was freely mobile and was seen lying along the posterior wall of maxillary sinus, and there were no signs of chronic sinusitis. The foreign body was removed with curved suction under 30°Hopkins sinoscope vision (Figures 3(a) and 3(b)) without complications. The postoperative period was uneventful, and his symptoms were relieved. The patient had an uneventful follow-up period of 2 months.

## 3. Discussion

The maxillary sinus anatomy and its relation to the roots of maxillary molars, premolars, and canines are in a way that many of the odontogenic infections and procedure can cause complications in the sinus. In addition, a thin floor of the maxillary sinus can lead to projection of the posterior teeth roots in some people [9]. In this case report, a dental burr was most likely introduced during the dental procedure. However, a medical practitioner must always suspect the possibility of foreign body in the maxillary sinus in a patient complaining of recurrent unilateral facial pain even if no history of dental procedure is elicited. In this case, an MRI was done in the patient, and this could have caused fatal complication due to the foreign body being dislodged to the brain or other vital structures.

Foreign bodies in the paranasal sinuses should be removed, even when they are asymptomatic to prevent mucosa irritations and reactions [1]. The pathophysiology of sinusitis caused by foreign bodies is still unclear. Tissue reactions and chronic irritation of the mucosa caused by foreign bodies could lead to a degree of ciliary insufficiency and then sinusitis [1].

A case report describes carcinoma of the maxillary sinus in a 48-year-old patient with a metal foreign body in the antrum [16]. Also, another case which was misdiagnosed as an ethmoid tumor but caused by a foreign body reaction to an amalgam filling was reported [12]. So, the prompt surgical intervention to remove the foreign body is expected to prevent the possible complications including sinusitis, mucosal cyst formation, foreign body granuloma, and persistent oroantral communication [3, 9].

Previously, the most common surgical technique used was the Caldwell–Luc procedure, which involves opening the anterior wall of the maxillary sinus [1, 2]. With advancement of imaging and endoscopic techniques, nasal and sinus endoscopic surgery is becoming the first-line approach for the removal of a foreign body from the maxillary sinus. If the foreign bodies are large enough, then their removal may not be easy by routine endoscopy [3]. In our case, endoscopic approach was applied.

## 4. Conclusion

Foreign bodies in the maxillary sinus are fairly uncommon. They usually enter sinus occurring during or secondary to a dental procedure. Whatever the nature of the foreign body might be, it must be removed to prevent chronic infections even if it is asymptomatic. Any patient presenting with recurrent unilateral facial pain or unilateral symptoms with/without previous history of sinusitis should raise the suspicion of a foreign body in the sinus regardless of any previous history of dental procedures.

We suggest that an endoscopic approach should be considered as the first-line option for removal of maxillary sinus foreign bodies. Endoscopic approach can be performed as an outpatient procedure and provides removal under direct vision with fewer complications and facilitates an early recovery.
